# The Greatest Enemy

**Published:** 1862-10

**Authors:** 


### THE GREATEST ENEMY

To any army is disease; it destroys three times as many as the sword. Knowing this, Miss Florence Nightingale abandoned all the advantages which culture, position, and an ample fortune gave her, for the purpose of devoting her whole attention to the preservation of her country's troops in the Crimean campaign. She found that the soldiers were dying from disease at a rate far more fearful than the most terrible devastations of the cholera in any civilized country; and before the war was over, there were fewer deaths from disease than among the most favored troops at home in England.

The three chief army diseases are, Fever, Diarrhea, Dysentery. A seasonable and possible care on the part of each soldier can effectually prevent three fourths of the cases of these diseases. All that is needed for such encouraging results is, that each man should understand what these diseases are, what causes them, and how he may avoid these causes. All this can be told without using a single medical term, in a manner which the most unlettered person can understand, and without the necessity of taking a single solitary grain or drop of any medicine whatever. And more, these diseases can be prevented in the vast majority of cases, with the means which a soldier has within his own power in a forest, on a prairie, or on a sand-bank.

Fever, of the ordinary kinds which prevail in camps, is preceded or accompanied in almost every instance by Constipation of the bowels, that is, by their failing to act once in each twenty-four hours. No man can possibly be well for a single week, who fails to go to stool every day. For a few days he may feel well, but before a week is ended he will most certainly be complaining in some way. Constipation and Costiveness are essentially the same thing, and seldom can exist for a single week at a time, without endangering life in many, and health in all.

Diarrhea is when the bowels act too often, from two to twenty times in twenty-four hours.

But it is not actual diarrhea unless a man feels after a passage as if he would like to sit down, feels weak, feels as if he would like never to get up again. The passages are large, and thin almost as water, there is no pain, no blood, and each passage gives relief, with an increasing disposition to sit or lie down; every human desire, every human ambition is comprised in the one privilege, to be able to lie down and rest; it seems to be a luxury to every muscle in the whole body, and there are upward of five hundred of them.

Dysentery, or Bloody Flux, on the other hand, is something between Costiveness and Diarrhea, something between a too infrequent and a too frequent action of the bowels, for it is a great and frequent desire to discharge something, but can not, except a little blood. The desire is intense and sudden, with a feeling as if it would give perfect relief, but when the effort is made, it produces a sensation which the ancients expressed by "Tormina" or torment.

*Costiveness* is less than one stool a day.

*Diarrhea* is more than one stool a day.

*Dysentery* is a constant desire, and yet an inability to stool.

Costiveness gives hard, bally, and scant stools.

Diarrhea gives thin, frequent, and copious stools.

Dysentery gives a frequent but unavailing desire to stool. See page 11, No. 33.

Costiveness may, or may not, give pain.

Diarrhea gives grateful relief.

Dysentery gives intense suffering, always and under all circumstances.

Costiveness may have a little blood.

Diarrhea never has any.

Dysentery always has blood; unless it has blood at almost every discharge, it can not be dysentery at all.

Costiveness unchecked, leads to a thousand different forms of disease, generally lasting a long time.

Diarrhea unchecked, leads to cholera and a speedy death.

Dysentery unchecked, leads to inflammation, and a death certain and painful, ending in mortification of the bowels.

But, as costiveness precedes fever, diarrhea, and dysentery, it is clear that if it is removed as soon as discovered, the chances for a soldier's exemption from every disease are incalculably increased. Hence the man who makes it his study and his care, his duty and his pleasure, to guard against constipation, or promptly remove it when by chance it occurs, not only avoids the risk of the ailments named, but acquires a vigor of health which repels a thousand ill influences, repels a thousand attacks from the causes of all other diseases; while if he was not entirely well when he entered the army, he will be pretty soon.

